# Belgian population norms for the EQ-5D-5L, 2018

**DOI:** 10.1007/s11136-021-02971-6

**Published:** 2021-08-18

**Authors:** Lisa Van Wilder, Rana Charafeddine, Philippe Beutels, Robin Bruyndonckx, Irina Cleemput, Stefaan Demarest, Delphine De Smedt, Niel Hens, Aline Scohy, Niko Speybroeck, Johan Van der Heyden, Renata T. C. Yokota, Herman Van Oyen, Joke Bilcke, Brecht Devleesschauwer

**Affiliations:** 1 Department of Public Health and Primary Care, Ghent University, Ghent, Belgium; 2 Department of Epidemiology and Public Health, Sciensano, Brussels, Belgium; 3 Centre for Health Economics Research and Modeling Infectious Diseases (CHERMID), Vaccine and Infectious Disease Institute (VAXINFECTIO), University of Antwerp, Wilrijk, Antwerp, Belgium; 4 School of Public Health and Community Medicine, The University of New South Wales, Sydney, Australia; 5 Institute for Biostatistics and Statistical Bioinformatics (I-BioStat), Data Science Institute, Hasselt University, Diepenbeek, Belgium; 6 Laboratory of Medical Microbiology (LMM), Vaccine and Infectious Disease Institute (VAXINFECTIO), University of Antwerp, Antwerp, Belgium; 7 Belgian Health Care Knowledge Centre (KCE), Brussels, Belgium; 8 Institute of Health and Society (IRSS), Université catholique de Louvain, Brussels, Belgium; 9 Department of Veterinary Public Health and Food Safety, Ghent University, Merelbeke, Ghent, Belgium

**Keywords:** Health-related quality of life, Health inequalities, Health interview survey, Health status, Multi-attribute utility instrument, EQ-5D, Visual analogue scale, Population norms

## Abstract

**Purpose:**

Health-related quality of life outcomes are increasingly used to monitor population health and health inequalities and to assess the (cost-) effectiveness of health interventions. The EQ-5D-5L has been included in the Belgian Health Interview Survey, providing a new source of population-based self-perceived health status information. This study aims to estimate Belgian population norms for the EQ-5D-5L by sex, age, and region and to analyze its association with educational attainment.

**Methods:**

The BHIS 2018 provided EQ-5D-5L data for a nationally representative sample of the Belgian population. The dimension scores and index values were analyzed using logistic and linear regressions, respectively, accounting for the survey design.

**Results:**

More than half of respondents reported problems of pain/discomfort, while over a quarter reported problems of anxiety/depression. The average index value was 0.84. Women reported more problems on all dimensions, but particularly on anxiety/depression and pain/discomfort, resulting in significantly lower index values. Problems with mobility, self-care, and usual activities showed a sharp increase after the age of 80 years. Consequently, index values decreased significantly by age. Lower education was associated with a higher prevalence of problems for all dimensions except anxiety/depression and with a significantly lower index value.

**Conclusion:**

This paper presents the first nationally representative Belgian population norms using the EQ-5D-5L. Inclusion of the EQ-5D in future surveys will allow monitoring over time of self-reported health, disease burden, and health inequalities.

**Supplementary Information:**

The online version contains supplementary material available at 10.1007/s11136-021-02971-6.

## Introduction

Health-related quality of life (HRQoL) refers to a person’s self-reported physical, mental, and social functioning and is an important outcome measure to support health care decisions [1]. Several instruments have been proposed to elicit HRQoL scores. One of the most commonly used generic multi-attribute classification systems accompanied by preference weights is the EQ-5D. The EQ-5D consists of a descriptive system of self-perceived health status along five dimensions (mobility, self-care, usual activities, pain/discomfort, anxiety/depression) and a visual analogue scale (VAS) which provides a self-rating of the general health status on a scale from 0 (worst imaginable state of health) to 100 (best imaginable state of health). The original EQ-5D descriptive system, the EQ-5D-3L, defined three severity levels (no problems, some or moderate problems, extreme problems/unable to) per EQ-5D dimension. In 2009, the more sensitive EQ-5D-5L was launched, with five severity levels (no problems, slight problems, moderate problems, severe problems, extreme problems/unable to) per EQ-5D dimension [2]. Translating the EQ-5D dimension scores into a single index value, or utility, requires an algorithm that can attach values to all possible EQ-5D health states. A utility ranges between 0 (death) and 1 (perfect health), but it can also include negative values for health states perceived worse than death [3]. Recently, the Belgian value set for the EQ-5D-5L has been developed based on health states preferences from the general population of Belgium [4].

In order to evaluate disease-associated loss in HRQoL for a given patient, reference data in terms of population norms, i.e., HRQoL data for the average person in the general population in a similar age and/or gender group, are required [5]. Several countries therefore now include generic HRQoL measures in their national health surveys to obtain such population norms [6–8]. The growing availability of population norms also offers an additional way of assessing and monitoring population health and health inequalities [9]. Indeed, with aging populations and the growing importance of non-fatal diseases, evidence-based public health policies require knowledge of population health in terms of morbidity and mortality [10].

In Belgium, National Health Interview Surveys (BHIS) have been organized since 1997 [11]. In the BHIS, information is collected on the health status, lifestyle, and health care utilization of a representative sample of the total Belgian population (including the regions of Brussels, Wallonia, and Flanders, which make up 10%, 32%, and 58% of the population, respectively). Since 2013, the EQ-5D-5L is included. We aim to present Belgian population norms for the EQ-5D-5L by age, sex, and region, and analyze its association with educational attainment using the most recent data from BHIS 2018, and compare changes in population norms between 2013 and 2018.

## Methods

### Belgian Health Interview Survey

BHIS data of 2013 and 2018 are used; however, we mainly focus on the BHIS 2018. The BHIS is a cross-sectional household survey, in which participants were selected from the national register through a multistage stratified sample of all persons officially residing in Belgium, without any restrictions on nationality. The sampling design involved a geographical stratification, a selection of municipalities within provinces, households within municipalities, and respondents within households. The net sample size of survey participants was 10,829 and 11,611 individuals in 2013 and 2018, respectively. The participation rate in the survey of 2013 and 2018 was 57.1% and 57.5%, respectively, at household level. The detailed methodology of the survey is described elsewhere [11]. In general, the BHIS collects information on health and well-being, health behavior and lifestyle, health care use, physical and social environment, and prevention.

### Measures

Data on age, gender, and educational attainment, collected through face-to-face interviews, were used in our analyses. Educational attainment was based on the highest level of education attained by the reference person of the household (i.e., person in charge of the administration of a household) or their partner, and regrouped into three categories: low (lower secondary education or less), intermediate (higher secondary education), and high (higher education).

### EQ-5D-5L

Self-perceived health status was assessed using the EQ-5D-5L and elicited via self-administered written questionnaires in four languages (Dutch, French, German, and English). For each possible EQ-5D-5L health state, a single index value can be derived based on country-specific value sets. We used the newly developed EQ-5D-5L value set for Belgium [4]. Possible index values range between − 0.532 (worst health state) and 1 (most optimal health state). We analyzed the EQ-5D VAS of the BHIS 2013 as the VAS was excluded in the BHIS 2018 due to practical limitations. The collection of EQ-5D data took place between January 2018 and January 2019.

Only individuals aged ≥ 15 years who were able to complete the self-administered written questionnaire were eligible (*n* = 7896 in 2013, *n* = 8837 in 2018). In total, the EQ-5D was completed by 77% of the eligible participants (*n* = 6061, mean age 48.4 years, 52% women) in 2013 and by 85% of the eligible participants (*n* = 7509, mean age 48.6 years, 52% women) in 2018. Sample characteristics of both waves are shown in Table 1.

**Table 1:** Characteristics of the study participants in 2013 (*N* = 6061) and 2018 (*N* = 7509), weighted

	2013	2018	*P*-value
Age, mean (SD)	48.4 (18.45)	48.6 (18.88)	< 0.001
15–24 years	11.0%	11.7%	< 0.001
25–44 years	32.7%	31.6%	
45–64 years	35.0%	35.1%	
≥ 65 years	21.2%	21.6%	
Sex			< 0.001
Female	52.0%	51.6%	
Male	48.0%	48.4%	
Educational attainment			< 0.001
Low	21.6%	16.8%	
Intermediate	33.7%	32.4%	
High	44.7%	50.8%	
Civil status			< 0.001
Single	26.6%	29.3%	
Married or legally cohabiting	55.6%	54.3%	
Widow(er)	7.7%	6.7%	
Divorced	10.1%	9.7%	
Region			< 0.001
Flanders	61.4%	58.6%	
Brussels	7.9%	9.0%	
Wallonia	30.7%	32.4%	

### Statistical analyses

Data were analyzed using the survey package for R 4.0.2 [12–14]. The design effects of the survey, i.e., the survey weights, clustering at household level, and regional stratification (henceforth referred to as “survey-weighted” analyses) were taken into account [11].

First, we presented the dimension scores by calculating the probability, stratified by sex (male, female), age (15–100 years), and region (Brussels, Flanders, Wallonia) of reporting each of the five levels per EQ-5D dimension. Next, results were dichotomized in problems (any problem) versus no problems per EQ-5D dimension. Finally, an aggregate outcome of problems on any EQ-5D dimension was reported. The EQ-5D’s ceiling effect (i.e., proportion reporting health state ‘11111’) was 39.0% and 35.2% in 2013 and 2018, respectively.

Then, the probability of reporting any problem per EQ-5D dimension was modeled using a survey-weighted logistic regression model. Eight models of increasing complexity were fitted, i.e., one intercept-only model, three models with one covariate (sex, age, region), three models with two covariates (sex + age, sex + region, age + region), and one model with three covariates (sex + age + region). For each model, all possible interaction terms were evaluated, and the model with lowest Akaike’s information criterion (AIC) was retained. To assess a possible non-linear association with age [15], age was included as a smooth function term using natural cubic splines. To select the optimal number of degrees of freedom, 10 models were run with degrees of freedom varying from 1 to 10, and the model with lowest AIC was chosen. Population norms for the probability of reporting any problem per EQ-5D dimension were then obtained by generating model-based predictions and corresponding 95% Wald-type confidence intervals (CIs), for any possible combination of age, sex, and region. Finally, an additional model was fitted to analyze the effect of educational attainment corrected for sex, age, and region. Goodness-of-fit of the latter models was calculated using the Cox–Snell pseudo R-squared.

The EQ-5D-5L index values and EQ-5D VAS scores were modeled using a linear survey-weighted regression model. Population norms by sex, age, and region were modeled using the same eight models and prediction approach as described above. As for the EQ-5D dimension scores, an additional model was fitted to analyze the effect of educational attainment corrected for sex, age, and region. Goodness-of-fit of the latter models was calculated using the R-squared statistic.

To evaluate significance of differences over time, we assessed whether the 95% CIs overlapped. Non-overlapping CIs were considered indicative of a significant difference, whereas overlapping CIs were considered inconclusive. Significance of differences between sex or region categories were assessed using regression models.

Finally, to assess the validity of the survey-weighted regression models using natural cubic splines (SVY-NS) to model (possible) non-linear age trends, we performed sensitivity analyses using alternative model definitions, i.e., survey-weighted regression models using fractional polynomials (SVY-FP), generalized additive mixed models using thin plate regression splines (GAMM), Beta one-inflated models using natural cubic splines (BEOI-NS), and Beta one-inflated models using fractional polynomials (BEOI-FP). The latter two models are of particular interest to model index values and VAS scores, since they allow taking non-standard response distributions into account, with fixed boundaries and a highly right-skewed distribution, favoring perfect self-perceived health. For each of the models, we calculated the sum of squared deviations and created residual plots, Q-Q plots, and plots of the fitted age-specific estimates against the observed age-specific means.

## Results

### EQ-5D-5L dimension scores

**Fig. 1:**
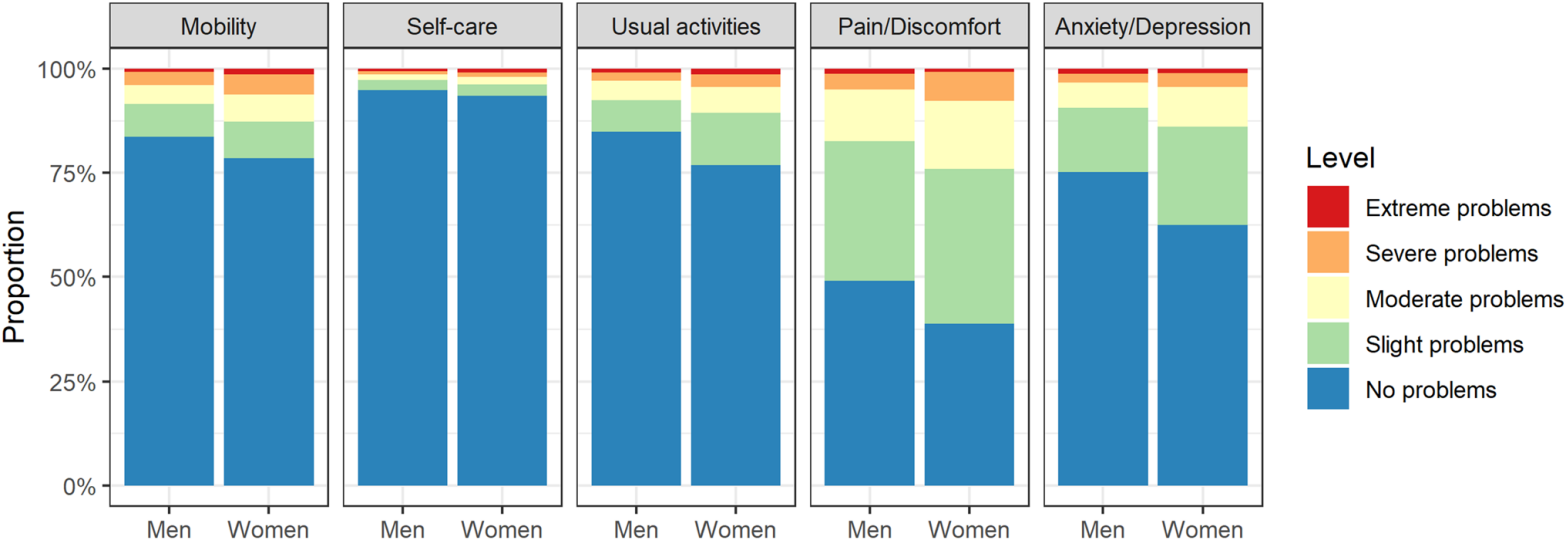
Survey-weighted probability of reporting problems on the five EQ-5D-5L dimensions, by sex, for the Belgian population aged 15 years and older, 2018

**Fig. 2:**
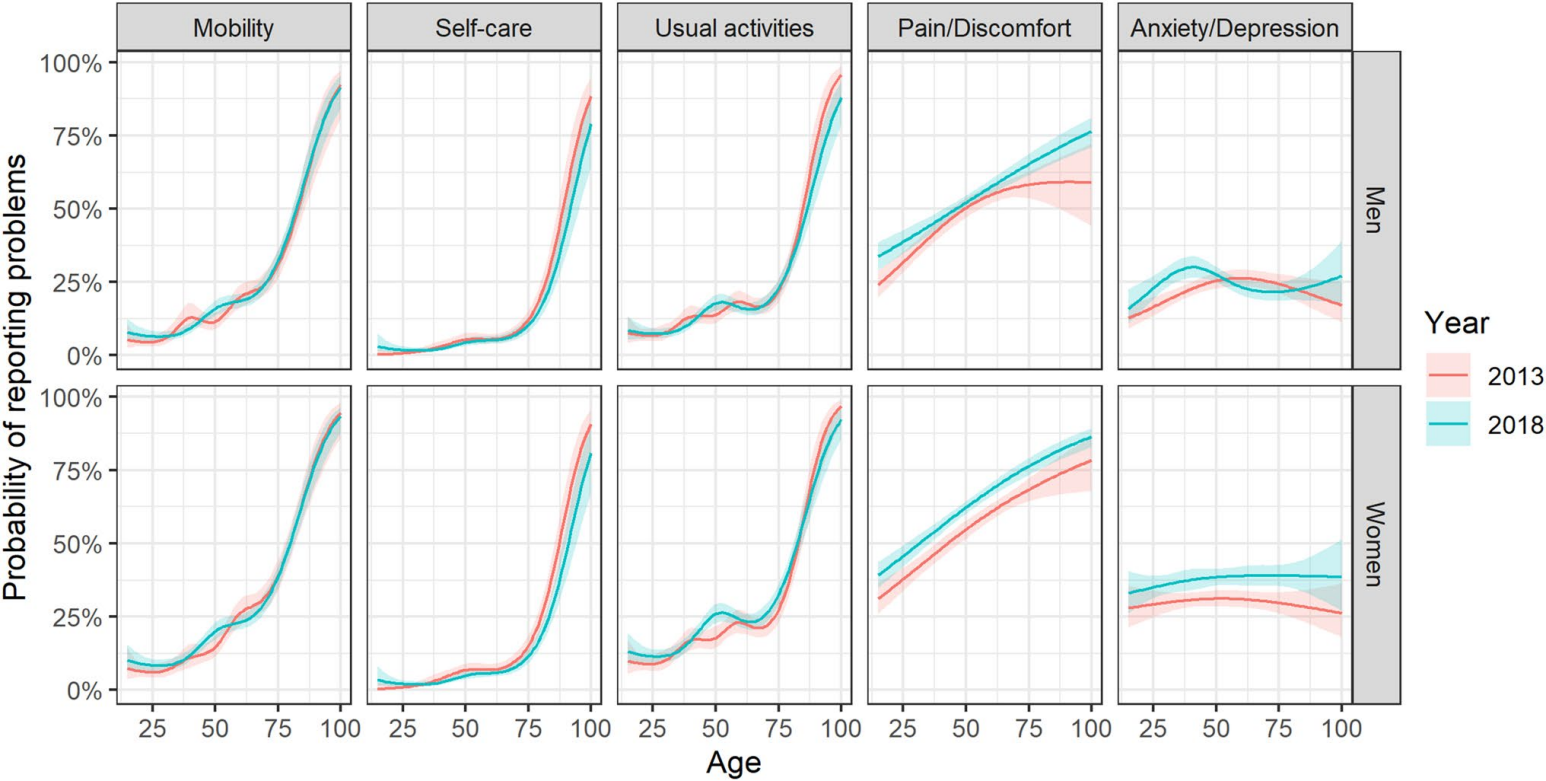
Population norms for the probability of reporting any problem per EQ-5D-5L dimension, by age and sex, for the Belgian population aged 15 years and older, 2013 vs 2018

More than half of the respondents reported problems of pain/discomfort (56%), followed by problems of anxiety/depression (31%), mobility (19%), usual activities (19%), and self-care (6%) (Fig. 1). A limited proportion of respondents reported extreme problems (3%), whereas 10% had severe problems and 24% had moderate problems at least on one of the five dimensions. Women reported problems more often than men on all dimensions and particularly on anxiety/depression (38% vs 25%, *P* < 0.001) and pain/discomfort (61% vs 51%, *P* < 0.001). For mobility, self-care, and usual activities, a sharp increase in reporting problems was seen after the age of 80 years. For pain/discomfort, the probability of reporting problems increases almost linearly with age. The probability of reporting problems of anxiety/depression did not show a clear association with age for women, while for men, a non-monotonic age distribution was estimated with a maximum between the ages of 35–55 and after the age of 75 (Fig. 2).

A significantly higher prevalence of reporting problems of mobility was estimated for Wallonia versus Flanders (23% vs 17%, *P* < 0.001). The prevalence of reporting problems of the following dimensions was significantly higher in Wallonia compared to both Flanders and Brussels: usual activities (23% vs 18% and 17%, *P* < 0.01), pain/discomfort (63% vs 53% and 52%, *P* < 0.001), and anxiety/depression (45% vs 23% and 39%, *P* < 0.001, *P* < 0.05); the prevalence of reporting problems of anxiety/depression was also significantly higher in Brussels compared to Flanders (39% vs 23%, *P* < 0.001) (https://github.com/brechtdv/popnorms; Annex 1).

The complete model, adjusted for sex, age, region, and educational attainment, revealed a significant (*P* < 0.001) higher prevalence of the mid and low categories of educational attainment among impaired health scorers on all dimensions except anxiety/depression (Table 2).

**Table 2:** Percentage of respondents and odds ratios (95% confidence interval) for reporting any problem per EQ-5D dimension and regression coefficients (95% confidence interval) for EQ-5D index value and EQ-5D VAS, adjusted for age, sex, region, and educational attainment

	EQ-5D dimensions										EQ-5D-5L index value		EQ-5D VAS score (2013)	
	Mobility		Self-care		Usual activities		Pain/discomfort		Anxiety/depression					
	%	OR (95% CI)	%	OR (95% CI)	%	OR (95% CI)	%	OR (95% CI)	%	OR (95% CI)		*β* (95% CI)		*β* (95% CI)
Sex														
Male	16	–	5	–	15	–	51	–	25	–	0.87	–	78.0	–
Female	22	1.32 (1.11;1.56)	7	1.10 (0.83;1.47)	23	1.62 (1.37;1.91)	61	1.48 (1.30;1.68)	38	1.84 (1.60;2.11)	0.82	− 0.04 (− 0.05;− 0.03)	76.3	− 1.27 (− 2.27;− 0.27)
Region														
Flanders	17	–	6	–	18	–	53	–	23	–	0.87	–	76.9	–
Wallonia	23	1.50 (1.23;1.84)	7	1.24 (0.90;1.70)	23	1.40 (1.15;1.70)	63	1.55 (1.32;1.81)	45	2.80 (2.39;3.29)	0.80	− 0.07 (− 0.09;− 0.05)	77.3	0.31 (− 0.91;1.52)
Brussels	19	1.48 (1.22;1.79)	5	1.33 (0.95;1.84)	17	1.13 (0.93;1.38)	52	1.04 (0.90;1.21)	39	2.26 (1.93;2.66)	0.84	− 0.04 (− 0.06;− 0.03)	77.9	− 0.14(− 1.50;1.22)
Education														
High	12	–	2	–	13	–	51	–	30	–	0.88	–	79.8	–
Mid	22	1.81 (1.47;2.25)	7	2.51 (1.74;3.63)	21	1.68 (1.37;2.07)	59	1.34 (1.14;1.58)	32	1.13 (0.96;1.34)	0.83	− 0.05 (− 0.06;− 0.03)	77.5	− 1.97 (− 3.14;− 0.80)
Low	34	2.02 (1.62;2.53)	14	3.36 (2.32;4.87)	33	2.16 (1.73;2.70)	67	1.38 (1.14;1.68)	35	1.14 (0.93;1.41)	0.75	− 0.09 (− 0.11;− 0.06)	70.6	− 6.24 (− 7.98;− 4.49)
Pseudo R^2^		0.12		0.07		0.09		0.07		0.07		0.12		0.10

Belgian Health Interview Survey, Belgium, 2018

### EQ-5D-5L index values

**Fig. 3:**
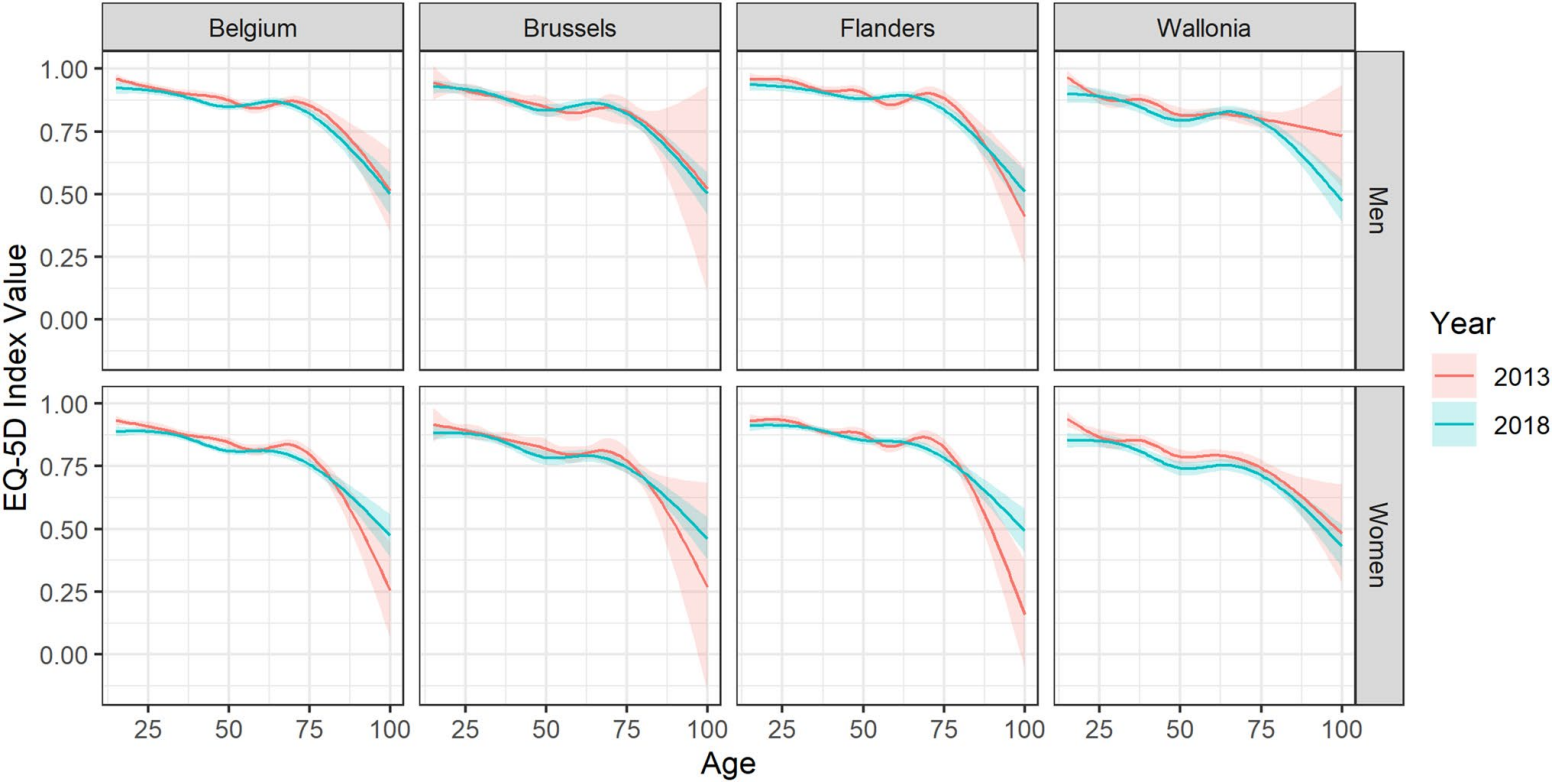
EQ-5D-5L index value population norms by age, sex, and region for the Belgian population aged 15 years and older, 2013 vs 2018

The average index value for the Belgian population aged 15 years and older was 0.84. The average score for men (0.87) was significantly (*P* < 0.001) higher than for women (0.82). Compared to Flanders (0.87), a significantly (*P* < 0.001) lower index value was estimated for Brussels (0.84) and Wallonia (0.80). Figure 3 shows an overall decreasing evolution of the index value by age, with a stronger decrease after the age of 70 years. The complete model showed a significant (*P* < 0.001) effect of educational attainment (Table 2), with an average index value of 0.88, 0.83, and 0.75 for the high, mid, and low educational attainment levels, respectively.

### EQ-5D VAS scores

**Fig. 4:**
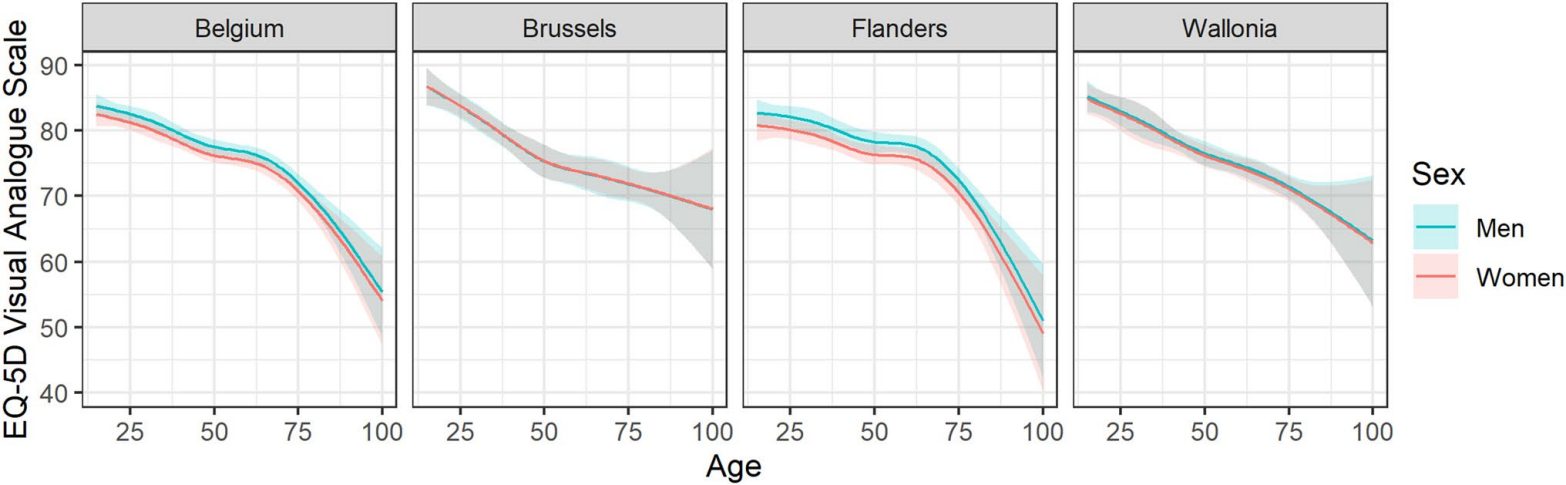
EQ-5D visual analogue scale (VAS) population norms by age, sex, and region for Belgium, population aged 15 years and older, 2013

In 2013, the average VAS score for the Belgian population was 77.1, with a significantly (*P* < 0.01) higher score for men (78.0) than for women (76.3). Figure 4 shows a monotonically decreasing evolution of the VAS score by age, without any clear inflection point. The complete model did not retain the significant differences between regions, but revealed a significant (*P* < 0.001) effect of educational attainment (Table 2), with an average VAS score of 79.8, 77.5, and 70.6 for the high, mid, and low educational attainment levels, respectively.

### Differences between 2013 and 2018

In general, evolution over time revealed a significant increase in the prevalence of reporting problems of pain/discomfort and anxiety/depression between the two time points. Moreover, the average index value for the Belgian population showed a significant decrease between 2013 and 2018.

### Validity of age models

The fits of the different models for age-specific HRQoL estimates are available on https://github.com/brechtdv/popnorms. The sensitivity analysis showed that the fits and point estimates of the SVY-NS, BEOI-NS, and GAMM models were relatively similar, except for older ages (80 and older) where the predictions differed more widely. The SVY-FP and BEOI-FP models provided the least flexible fits, often resulting in worse goodness-of-fit.

### Availability of population norms

Population norms by age, gender, region, and all possible combinations thereof are available in the Supplementary Materials (2018: Appendix 1; 2013: Appendix 2). To facilitate access to the results, we have also made them available as an R package (https://github.com/brechtdv/EQ5D.be).

## Discussion

Based on the BHIS 2018, we estimated nationally representative population norms for the EQ-5D-5L by sex, age, region, and educational attainment. In addition to being indispensable for cost-utility analyses, as currently recommended by Belgian guidelines for economic evaluations in health care [16], these population norms also present novel insights on the self-perceived health status of the Belgian population. These results may serve as a benchmark for follow-up in future BHIS.

Consistent with the evidence regarding population norms, our results show that female gender and older age were significantly associated with lower self-perceived health status. We identified a gender association with both the EQ-5D dimensions, particularly on anxiety/depression and pain/discomfort, and the EQ-5D index values. The pattern of women having a higher prevalence of reporting problems as well as having lower index values than men has already been observed in many countries [17–19]. The significant reduction with age for the index values is also consistent with the population norms from other countries [20–23]. Among the EQ-5D dimensions, problems of mobility, usual activities, and self-care showed a clearer-cut direct association with increasing age (prevalence < 10% in teenagers versus > 50% in octogenarians). This observation is in line with the increasing prevalence of disability at older ages previously documented in Belgium [24].

We found that higher education was significantly associated with a lower prevalence of reporting problems (except for anxiety/depression) and was related to higher index values. These results emphasize the socioeconomic inequalities in health in terms of educational attainment, which has been shown in Europe for decades [22]. According to region, our results show a significant higher prevalence of reporting problems on mobility, pain/discomfort, and anxiety/depression and significantly lower index values for people living in Brussels or Wallonia compared to Flanders. The associations with region on the other EQ-5D dimensions were less pronounced. Explanations for regional effects should be searched in socioeconomic characteristics, differences in lifestyles and in inside/outside air pollution, and differences in health policies and in health care management [25]. In addition, differences can also be attributed to the language version of the EQ-5D-5L used across the three regions (Flanders: 95.3% Dutch version, Brussels: 95.9% French version, Wallonia: 97.2% French version).

The Belgian population norms for the EQ-5D-5L appear to be consistent with those from other European countries, which also found that problems with pain/discomfort had the highest prevalence and problems with self-care had the lowest prevalence. The prevalence of reporting problems with pain/discomfort in the BHIS is, however, higher than the prevalence reported in Spain (28%) [26], Germany (32%) [27], England (42%) [28], Italy (43%) [29], and the Netherlands (49%) [30]. Only Poland reported a comparable high prevalence of pain/discomfort (52%). The second highest prevalence was reported in problems with anxiety/depression with nearly one in four Belgians reporting these problems. Among other European countries, the prevalence of reporting problems of anxiety/depression in Belgium was lower than in Poland (42%) [31] and Italy (38%) [29], but reported to be higher than in Spain (16%) [26], Germany (18%) [27], the Netherlands (21%) [30], and England (24%) [28]. Several possible explanations exist explaining the difference in prevalence between countries, such as different sociodemographic structures and cultural differences in health perceptions [23, 32]. The EQ-5D index values are furthermore affected by differences in valuation methods, which reflect the preferences of the country of elicitation [33, 34]. Indeed, the average index value (0.84) was lower than in other European countries, where it ranged from 0.87 (the Netherlands) to 0.92 (Italy) [26, 31, 30, 31, 35]. The Belgian VAS score (77.1) was lower than in Italy (78.2) [29], England (78.4) [28], the Netherlands (80.6) [30], and Germany (84.3) [27], but higher than in France (73.4) [35] and Spain (75.7) [26].

It should be acknowledged that the significant differences in EQ-5D index values or VAS scores, e.g., between sexes or across countries, may not be meaningfully different from each other. Indeed, interpreting the relevance of differences is not straightforward. Several authors have attempted to estimate the smallest difference in utility scores that an individual can recognize and appreciate, often termed the minimal important difference (MID) [36]. Walters and Brazier [37] estimated a mean MID for the EQ-5D of 0.074 [37]; likewise, Pickard et al. [38] estimated an MID for VAS scores of 7 [38]. MID values however depend on the characteristics of the study populations they were derived from, as well as the computational methods used to calculate the values. A review showed that MID values for the EQ-5D ranged widely, from 0.03 to 0.52 [36]. Furthermore, what constitutes an important difference is necessarily context-dependent, as even small differences in quality-adjusted life years may lead to large differences in incremental cost-effectiveness ratios. Interpreting cross-country differences in index values is further hampered by the fact that the values are the result of both the dimension scores and the value set used [39].

Our estimates represent the first Belgian HRQoL population norms by age, sex, and region, and the first based on the EQ-5D-5L. Earlier Belgian population norms were based on smaller sample sizes and used the EQ-5D-3L. König et al. [40] estimated population norms based on 2411 respondents who completed the EQ-5D between 2001 and 2003 [40], resulting in an average EQ-5D-3L index value of 0.883 [17]. Interestingly, only 6.6% of their respondents reported problems with anxiety/depression, compared to 31% in the BHIS. This discrepancy may be due to the more sensitive and precise nature of the EQ-5D-5L as compared to the EQ-5D-3L [41]. More recently, Bilcke et al. [42] used a representative sample of 1774 persons surveyed between 2010 and 2011 to estimate Flemish population norms for all ages and to explore potential associations of these health status measures with several potential determinants [42]. As in our study, older age and lower education was significantly associated with lower self-perceived health status, however, no association with sex was found, which contrasts the existing literature. Bilcke et al. [42] also found significant associations with smoking behavior, pet ownership, past experience with severe disease (in oneself or family members), working or having worked in health care, household size, and people aged 60 + years living on their own [42].

Modeling health status responses is not straightforward. The index value and VAS score have a non-normal distribution, with fixed boundaries and a highly right-skewed distribution, as the majority of the general population reported good self-perceived health. Basu and Manca [43] proposed the use of a (one-inflated) beta regression model to better capture these idiosyncrasies [43]. However, Bilcke et al. [42] noted that this approach would result in a limitation in the type of models that can be fit, as complex models with a beta error distribution are currently not readily available in standard statistical software [42]. In our case, the ideal model would allow taking three characteristics into account, i.e., the survey design, the non-linear age relationships, and the non-standard response distributions of the index values and VAS scores. Since such a model is not available in current statistical software, we had to resort to models that do not meet all criteria, thereby making trade-offs between good estimations of point estimates versus good estimations of standard errors. We used a survey-weighted linear regression model with natural cubic splines to model the age-specific index values and VAS scores, which allowed to properly take the survey design into account and to model non-linear age relationships, but used a Gaussian response distribution instead of a theoretically more suitable Beta one-inflated distribution [44]. To assess the possible impact of this limitation, we performed sensitivity analyses using alternative model definitions, including Beta one-inflated models. These sensitivity analyses revealed similar point estimates across model definitions, reinforcing the use of a survey-weighted linear regression model, yielding both accurate point estimates and standard errors. Differences across models mainly seemed to occur for the ages ≥ 80 years. This is a result of the lower sample size at these older ages, as also reflected by the larger confidence intervals of the individual models. Estimates of HRQoL for the very old based on the BHIS should therefore be treated with caution.

One of the limitations of the current study is that it considered only four potential determinants of self-perceived health status: age, sex, region, and educational attainment. All four of these turn out to be significant; however, despite the addition of these important covariates and the flexible, non-linear association with age, a lot of the variability has been left unexplained, resulting in relatively low goodness-of-fit estimates and trends in the diagnostic plots. Nonetheless, for the purpose of health economic evaluations, age, gender, and region are by far the most relevant categories to distinguish for population norms informing cost-effectiveness in the Belgian context. The main objective of this study was to provide population norms by age, gender, region, and combinations thereof. Future analyses will focus on explaining HRQoL by exploring associations with other determinants, including chronic disease status, and thus finding the model with the most optimal goodness-of-fit.

Certain limitations of the BHIS also apply to this study. First, although the survey included people in nursing homes, it excluded specific institutionalized populations such as prisoners and people residing in mental hospitals. Although these populations only represent a small fraction of the total population (less than 0.1%, of which more than 90% are men), they are likely to report impaired health status due to higher rates of physical and mental problems [45]. The results may also be biased toward a more healthy population by excluding participants aged ≥ 15 years for whom a proxy interview was conducted because they were not able to answer themselves. These participants are usually in worse health. In general, BHIS participation itself may be affected by a healthy volunteer effect. Second, the BHIS did not collect EQ-5D information from children under the age of 15 years. Estimating and interpreting HRQoL in children is not straightforward, as it often relies on responses from proxies. Wille et al. [46] proposed a youth version of the EQ-5D (EQ-5D-Y), which can be applied to children aged 8–15, either directly or via a proxy [46]. However, they also noted that existing EQ-5D value sets could not be assumed appropriate preference weights for the EQ-5D-Y. A third limitation of the BHIS, as in many other health surveys, is the potential bias due to educational differences in survey participation and the willingness and ability to answer the self-administered questionnaire [47]. To account for unequal representation of educational strata, Van der Heyden et al. [48] recommended including education in the calculation of post-stratification weights [48], which may be explored in future iterations of the BHIS; however, it should be kept in mind that weighting does not solve a potential selection bias. A final limitation is the exclusion of the EQ-5D VAS in the BHIS 2018 due to practical restrictions. Therefore, evolution over time for the EQ-5D VAS could not be assessed, although this could be of interest.

Future studies may build on the results presented here to gain additional insights in the self-reported health of the Belgian population by characterizing and analyzing inequalities in HRQoL and losses in HRQoL associated with self-reported chronic disease status or health determinants [49, 50]. Furthermore, linking the BHIS with mortality data would allow quantifying quality-adjusted life expectancy, as a summary measure that encompasses both quantity and quality of life [51]. Finally, by continuing to include the EQ-5D in future BHIS, it will become possible to further monitor self-perceived health status and inequalities therein over time.

## Conclusion

We estimated, to the best of our knowledge for the first time, nationally representative HRQoL population norms for the Belgian population by age, sex, and region using the EQ-5D-5L. Older age, female gender, Brussels or Walloon residents, and low educational attainment were significantly associated with a lower self-perceived health status. Inclusion of the EQ-5D in future BHIS will allow monitoring self-reported health, disease burden, health inequalities, and quality-adjusted life expectancy.

## Supplementary Information

Below is the link to the electronic supplementary material.

Supplementary file1 (XLSX 392 kb)

Supplementary file2 (XLSX 344 kb)

## Data Availability

To facilitate access to the results, we have made them available as an R package (https://github.com/brechtdv/EQ5D.be). Access to micro data of the BHIS can be requested via https://his.wiv-isp.be.
